# MEG Neural Decoding Pipeline: The Issues Residing Within The Data and Methods to Improve Your Decoding Accuracy

**DOI:** 10.1007/s10439-026-04093-x

**Published:** 2026-04-09

**Authors:** Dmitry Patashov, Li Liu, Jion Tominaga, Kai Nakajima, Hiroki Miyanaga, Shoji Tsunematsu, Takanori Kato, Keita Tanaka, Hiromu Sakai

**Affiliations:** 1 Waseda Research Institute for Science and Engineering, Waseda University, Tokyo, Japan; 2 Center for Brain Science, RIKEN Institute, Saitama, Japan; 3 Graduate School of Advanced Science and Engineering, Waseda University, Tokyo, Japan; 4 Faculty of Science and Engineering, Waseda University, Tokyo, Japan; 5 Faculty of Human Sciences, Waseda University, Tokyo, Japan; 6 Sumitomo Heavy Industries, Tokyo, Japan; 7 Sumimec Engineering, Niihama, Japan; 8 Sumitomo Heavy Industries, Yokosuka, Kanagawa Japan; 9 Department of Science and Engineering, Tokyo Denki University, Saitama, Japan

**Keywords:** Biomagnetism, Signal processing, Neural decoding, Machine learning, Data augmentation, CA, MEG, EMD, PCA

## Abstract

This study suggests a new analysis pipeline of MEG data, uniquely designed for neural decoding of small-sized datasets. It combines classic methods that assume stationarity of the data together with non-stationary methods to compensate for the distortions created by the classic approach. Popular Fourier-based methods are applied in a classic fashion, followed by additional filters using empirical mode decomposition and principal component analysis to further clean the data. An automated approach for epoch rejection is proposed as well. In this work, we propose a novel approach for data augmentation. Unlike most other solutions, combinations’ averaging technique can be used on real data rather than synthetic one, making it more reliable from the neuroscientific point of view. It is also shown that this approach does not create any unnatural patterns within the augmented data. The proposed approach allows for application of machine learning algorithms on small-sized datasets. This broadens the list of available analyses for datasets with limited number of recorded examples. An image naming task was used for in-subject neural decoding estimations. In this work, we propose and compare four different machine learning designs. It is shown that a careful selection of the used channels, reduction of the feature dimensions, and averaging of the recorded epochs may significantly increase the accuracy of neural decoding.

## Introduction

### Neural Decoding of MEG Data

Neural decoding is one of the multivariate pattern analyses of neural activities. Its goal is to decode the information within the recorded neural activity to predict the kind of stimuli received or behavior conducted by a subject. For example, relying solely on the recorded brain activity, being able to specify what is depicted in the image that the subject was shown. Neural decoding is important for understanding the neural activity related to the input or the output of the human brain. It can help us better understand the mechanisms that brain uses for encoding, storage, and retrieval of information [1–3]. Neural decoding also attracts attention of Brain–Machine Interfaces (BMI) researchers. There are countless applications in which BMI plays a big role [4, 5]. For example, an accurate neural decoding system may help communicate with people who are otherwise unable to do so (e.g., locked in syndrome). There are multiple neuroimaging techniques that can be used for neural decoding, among which are the following: functional Magnetic Resonance Imaging (fMRI), functional Near-Infrared Spectroscopy (fNIRS), Electroencephalography (EEG), Magnetoencephalography (MEG), etc. Each technique has its own unique properties, strengths, and weaknesses. For example, although fMRI can provide a very high spatial resolution, its temporal resolution is relatively low. It is also not portable and does not allow much movement. fNIRS on the other hand is portable and allows for relatively free, natural movements to be made. However, it can only record the hemodynamic activity from superficial layers of the brain. Non-invasive EEG has relatively high temporal resolution, but its spatial resolution is limited. Invasive EEG on the other hand may achieve high spatial and temporal resolutions, resulting in improved accuracy of decoding. It was shown before that a recording from surgically implanted electrode exhibited successful decoding with high accuracy and fine time resolution [6, 7]. However, the use of invasive methods is limited and may face difficulties in some practical applications. It also does not normally provide a full head measurement as the electrodes are usually placed only around the site that requires a medical intervention, thus not allowing the examination of the remote brain regions. Non-invasive methods attract much attention for such reasons. MEG, being a non-invasive method which also achieves both high temporal and good spatial resolutions [8, 9], is becoming a popular choice for neural decoding. Using Superconducting Quantum Interference Devices (SQID), MEG measures subtle changes of magnetic fields created by the electric activities of neuron populations. Since magnetic fields are not influenced by the conductivities of tissues surrounding the human brain, they provide more reliable information about the sources of neural activities. Combined with its high temporal resolution, MEG is thus regarded as a suitable tool to identify the sources of rapidly emerging neural activities using neural decoding analysis. Just like any other method, MEG has its own limitations. Noise and variabilities across trials and among participants are relatively high [10]. Careful preprocessing of the relatively large number of data to remove noise and reduce variability is critical for successful decoding, but it requires a relatively large number of subjects or task repetitions [11–13]. Considering that in the field of neuroimaging, it is common to have very limited datasets, it is even more important to develop solutions that can successfully decode the neural activity from a limited number of samples. Unfortunately, the number of studies using MEG data for neural decoding is limited compared to fMRI. It is yet to be established on what is the best way of noise removal among possible options. One of the remaining goals is the creation of a reproducible pipeline that would contribute to the development of safe and scalable, non-invasive methods of decoding [14].

In this work, we propose a new pipeline for MEG data processing and analysis, uniquely designed for neural decoding, aiming to improve the interpretability of the brain signals via decoding accuracy. It combines classical methods together with relatively new approaches, making it more resistant to potential data distortions. A novel approach for data augmentation is proposed to supplement the need for large datasets. Reduction of feature dimensions, selection of channels, and ways of averaging epochs are also proposed to yield an increased accuracy of decoding. We hypothesize that channel selection procedure may reduce the model overfitting effects and reduce linear dependencies within the feature vectors, which in turn should improve the classification accuracy. It is well known that averaging of repeated events reduces white noise within the data, making it cleaner and more interpretable. Therefore, we believe that augmenting the data using the averaging approach should improve the decoding accuracy.

### Stationarity

Stationarity refers to the stability of the statistical behavior of a process. There are three main stationarity categories: Strict Sense Stationary (SSS), Wide Sense Stationary (WSS), and Non-Stationary (NS). In mathematics, SSS is defined as invariance of all finite-dimensional distributions under time shifts, whereas WSS requires a constant mean and an autocovariance that depends only on the time lag. Because we observe a single realization of the underlying stochastic process, stationarity is assessed in practice via windowed (time-local) estimates of mean, variance, and autocorrelation [15]. Since in reality it is nearly impossible to find a natural process that is SSS, let us concentrate on a more realistic definition, which is WSS. It requires only the first and second-order moments (henceforth first and second statistical moments), $M_{1} \left( t \right)$ and $M_{2} \left( {t_{1} ,t_{2} } \right)$, to be time-shift invariant. Nonetheless, even this condition is rarely met in natural processes. Most natural processes usually have a time dependency in at least one of the two first statistical moments, making them NS.

MEG data, just like most of neuroimaging data, are typically NS [16]. The majority of neuroimaging systems introduce a low-frequency interference called drift. This interference is a stochastic process associated with the first statistical moment. The presence of a drift within the data means that the first statistical moment is time dependent and therefore the data are NS. That said, there are many known ways to reduce the effects of drift, making the first statistical moment constant or nearly constant (the variations in time are insignificant). The main issue within the neuroimaging data, however, mostly comes from the time-dependent second statistical moment. Brain processes are very complex, having variations in their amplitude and frequency patterns that tend to change with time. Basically, this variability indicates that the second statistical moment is time dependent. Reducing these effects to make the second statistical moment constant is very complicated and in many cases is not feasible at all. Not to mention that unlike drift, this time dependency-inducing phenomena may be important in understanding the neural patterns. Therefore, even if this time dependency can be removed, it might be counter-productive in terms of our goals. As there is no practical way to assure stationarity of the recorded data, it is very important to carefully design the processing pipeline. Most of the commonly used methods assume stationarity of the process they are applied to. When these methods are applied to NS data, they introduce distortions into the data, making it less reliable. Although it is usually impossible to fully avoid any distortions, by carefully designing the processing pipeline, such distortions can be reduced.

### Combination’s Averaging

Grand Averaging (GA) is a very common practice in neuroscience [17–19] as it significantly reduces the white noise within the recorded signals, given a sufficient number of examples (epochs). This process also partially suppresses time-dependent and localized colored noise. Although it is a very good method for analyzing the patterns of the recorded brain activity, it is not very suitable for Machine Learning (ML) procedures, most of which require a large number of examples to train and test a model on. For ML procedures, one can divide the examples into smaller sub-groups and perform averaging within each sub-group (sub-GA) [20]. This would reduce the noise and still retain a number of examples equal to the number of the selected sub-groups. Although such an approach could be used for ML purposes, it still requires a large number of examples. Without a sufficient number of epochs, one may not have enough sub-groups for the ML or may not have enough epochs within each sub-group to make a substantial difference in noise level reduction. Therefore, in studies where the number of epochs is relatively low, sub-GA approach may be inapplicable as well.

In this work, we propose a method called Combination’s Averaging (CA). This method of data augmentation can be applied to relatively small datasets and is suitable for ML models. Most approaches of data augmentation for neuroimaging data concentrate on synthetic datasets [21, 22]. Some approaches use the existing data to try extrapolating the data into new examples [23, 24]. CA concentrates on real data augmentation. Instead of creating new data that may or may not represent the real one, it reuses the real data in a way that allows to increase the data size. In turn allowing for a reliable analysis of subjects’ brain activity. This approach reduces the white noise within the data and at the same time, depending on the size of the original dataset, may significantly increase the number of examples that can be used in ML models. The CA’s design allows for it to be applied in places, where GA and sub-GA are inapplicable or impractical.

## Methods

### Subjects

Ten right-handed Japanese native speakers, age 18 or older, participated in the experiment. Data collected from three of the subjects were discarded due to some technical issues during the recording. Remaining seven subjects’ (six male and one female, average age 23) data were used in this study. The participants were recruited from Waseda University and Tokyo Denki University. All participants had normal or corrected-to-normal vision and reported no history of neurological or language disorders. The study was approved by the Ethics Review Committee on Research with Human Subjects of Waseda University on 7^th^ of May 2020 (ID: 2020-041). All researches were performed in accordance with guidelines and regulations of the Ethics Review Committee and in accordance with the Declaration of Helsinki. Prior to the experiment, each subject was informed of the details of the experiment and signed a written consent drafted by the Ethics Review Committee.

### Experimental Paradigm

**Fig. 1:**
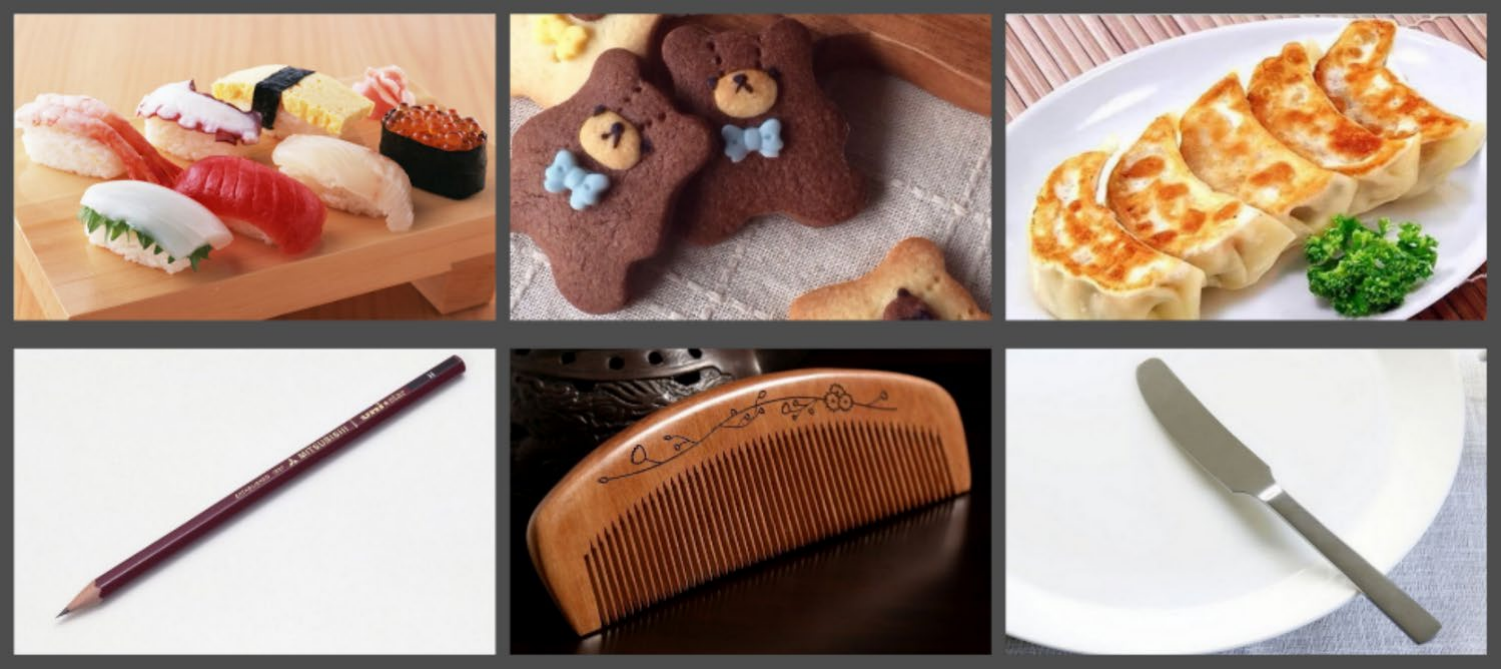
Images that were used as visual stimuli. from left to right, top to bottom: sushi, cookies, Gyoza, pencil, comb, and knife

The subjects were placed inside the Sumitomo Heavy Industries Magnetoencephalography (SHI-MEG) machine [25–27] in an upright standing position (for detailed information, please refer to the section 1 of the Supplementary Materials). To reduce leg fatigue, a leg-support device (ArchelisFX, Archelis Inc.) was used during the experiment. A delayed picture naming task was used in this experiment. The participants were asked to name the items in the images. The visual stimuli (i.e., images) were presented on the screen in a randomized order followed by a specific cue for response initiation. Six pictures: sushi, gyoza, cookies, comb, knife, and pencil were selected from two categories (food and tools) [Figure 1].

**Fig. 2:**
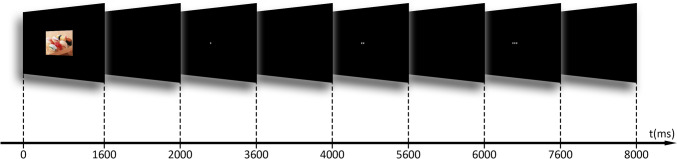
Example of a single trial within the experimental design. Horizontal axis is the time in milliseconds. The dashed lines indicate the time of the screen transitions between the stages of the trial. The images show which stage of the trial appeared on the screen at each time point

The linguistic expressions corresponding to the images were matched with their length (number of mora) and frequency for each category. The stimuli were visually presented on a nonmagnetic projection screen inside the MEG machine chamber. Each trial consists of four steps: Step 1: stimuli presentation (at 0ms); Step 2: the ‘*’ cue presentation (at 2000ms); Step 3: the ‘**’ cue presentation (at 4000ms); and Step 4: the ‘***’ cue presentation (at 6000ms). At 8000ms the whole sequence would restart. The instructions for step 1 were to silently observe the image. For step 2, to imagine an action involving the item from step 1 (eating the food or using the tool). For step 3, to name the item silently. For step 4, to overtly pronounce the name of the item. The trial design is illustrated in Figure 2. Each session was 10 minutes long. Each subject performed 4 sessions during the experiment, making a total number of repetitions per image to be 50. After each session a short break was introduced, and subjects were allowed to exit the machine. They were allowed to rest until they were ready to proceed. If any subject wished to exit the machine in the middle of a session, the session was terminated, and the data of that session were discarded. The subjects were allowed to redo the session and complete the experiment if they wished to continue, otherwise, the subject’s experiment data were discarded completely. In total, participants spent no longer than an hour performing the experiment. The dataset can be found on Zenodo repository [28].

### Tools

- Processing device was Dell Alienware Area 51 Laptop.
  - CPU: Intel Core Ultra 9 275HX 2.7, 24 Cores, 36MB cache, 5.4 GHz.
  - RAM: DDR5, 6400MT/s, 64GB.
  - GPU: NVIDIA GeForce RTX 5090, 24GB, GDDR7.
  - SSD: M.2 NVMe PCIe Gen5, 2TB.
- Processing and analyses were done using Anaconda 3 with Python 3.11.
- Experiment design was done using PsychoPy [29].
- Data management, and some standard operations were done using MNE toolbox [30].
- The functions from scikit-learn library were used as cores for our ML designs.
- Anaconda emd package 0.7.0 was used for signal decompositions.

### Preprocessing

#### Spectral Filtering

**Fig. 3:**
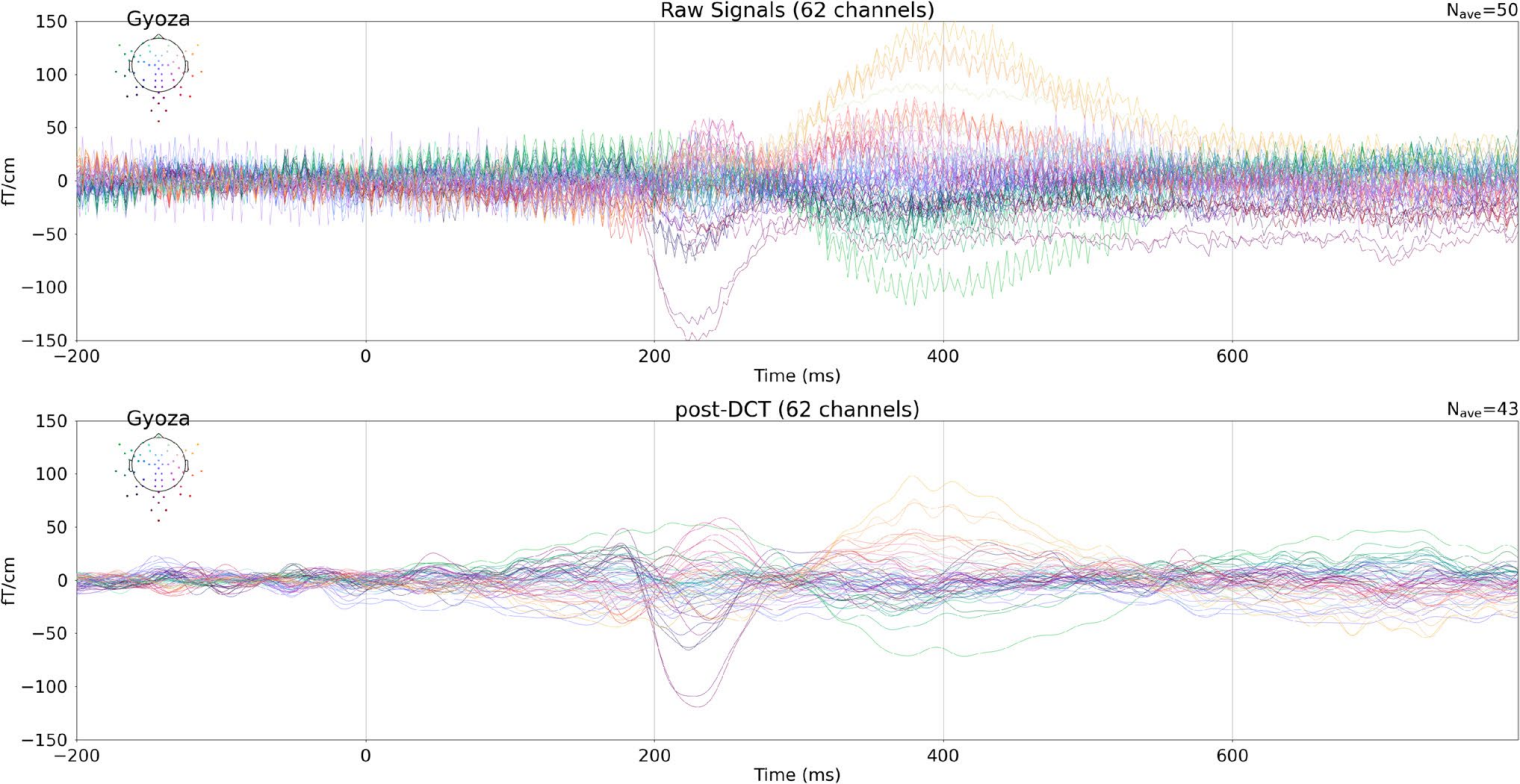
Example of the effects produced by the filters. Upper panel shows grand average of a single type of event (Gyoza) using raw data. Lower panel shows grand average of a single type of event (gyoza) using post-DCT filter data. Bad epochs were dropped before averaging. The plots show 62 channels (all the good channels) color-coded to match the placement locations as shown in the upper left corner of each sub-plot. Gyoza refers to the image that was shown in this specific event. Upper right corner shows N_ave_ value, which refers to the number of averaged epochs in that sub-plot

The loading, handling, resampling, and Band Pass Filtering (BPF) of the MEG data were done using MNE toolbox [30]. Two channels were discarded due to strong noise. Collected data were resampled to 500Hz. Our MEG device has substantial noise in the low-frequency range, below 1Hz, and in the high-frequency range past 50Hz. For our purposes and due to the system limitations, we have decided to concentrate on the frequency range mainly containing the bands from Delta to Beta. Our designed band-pass filtration process includes several steps in order to reduce the distortion of the data. The first step of the procedure uses BPF with a pass-band of 0.1 to 40Hz [31]. In principle, classic BPF assumes stationarity, therefore introduces distortions into the data. Effect size of these distortions is the strongest near the cutoff frequencies. Therefore, to reduce the distortions near border frequencies and the noise below 1Hz, two additional filters were used. Discrete Cosine Transform (DCT) was used for High Pass Filtering (HPF) with cutoff frequency of 1Hz, resulting in a pass-band of 1 to 40Hz [Figure 3]. As for high-frequency noise and distortions, Empirical Mode Decomposition (EMD) [32, 33]-based filter was used post-epoching procedure.

#### Epoching

Stimuli channels were produced by StimTracker that was connected to an A\D converter of the MEG machine. The signals were converted to analog for the system’s synchronization purposes. The resulting analog stimuli channels, marking the onsets of the trials, had non-instant transitions, overshooting and undershooting near transition locations (time points where the signal’s state changes between on and off) and some oscillations along the signals. When using MNE toolbox [30], epoch detection was inconsistent. In some cases, the number of detected epochs exceeded the number of actually recorded ones, in others, only some of the epochs were detected. To solve this issue, we binarized the stimuli channels removing any fluctuations and preserving only “high” and “low” values, with an instant transition in between. Following this procedure, MNE toolbox [30] was successful in epoching the data correctly. The extracted epochs contained 200ms pre-stimuli and 800ms post-stimuli recording. In total, each epoch was 1s long. Outlier epochs were detected and removed using an epoch-outlier detection procedure designed in this study. It tested the variability of the signals in time, across epochs. The epochs that had an unusual variability were discarded. The detailed mathematical formulation can be found in section 2 of the Supplementary Materials. The proposed procedure has allowed for accurate and robust epoch-outlier detection that is unaffected by the poorly recorded channels.

#### Empirical Mode Decomposition

**Fig. 4:**
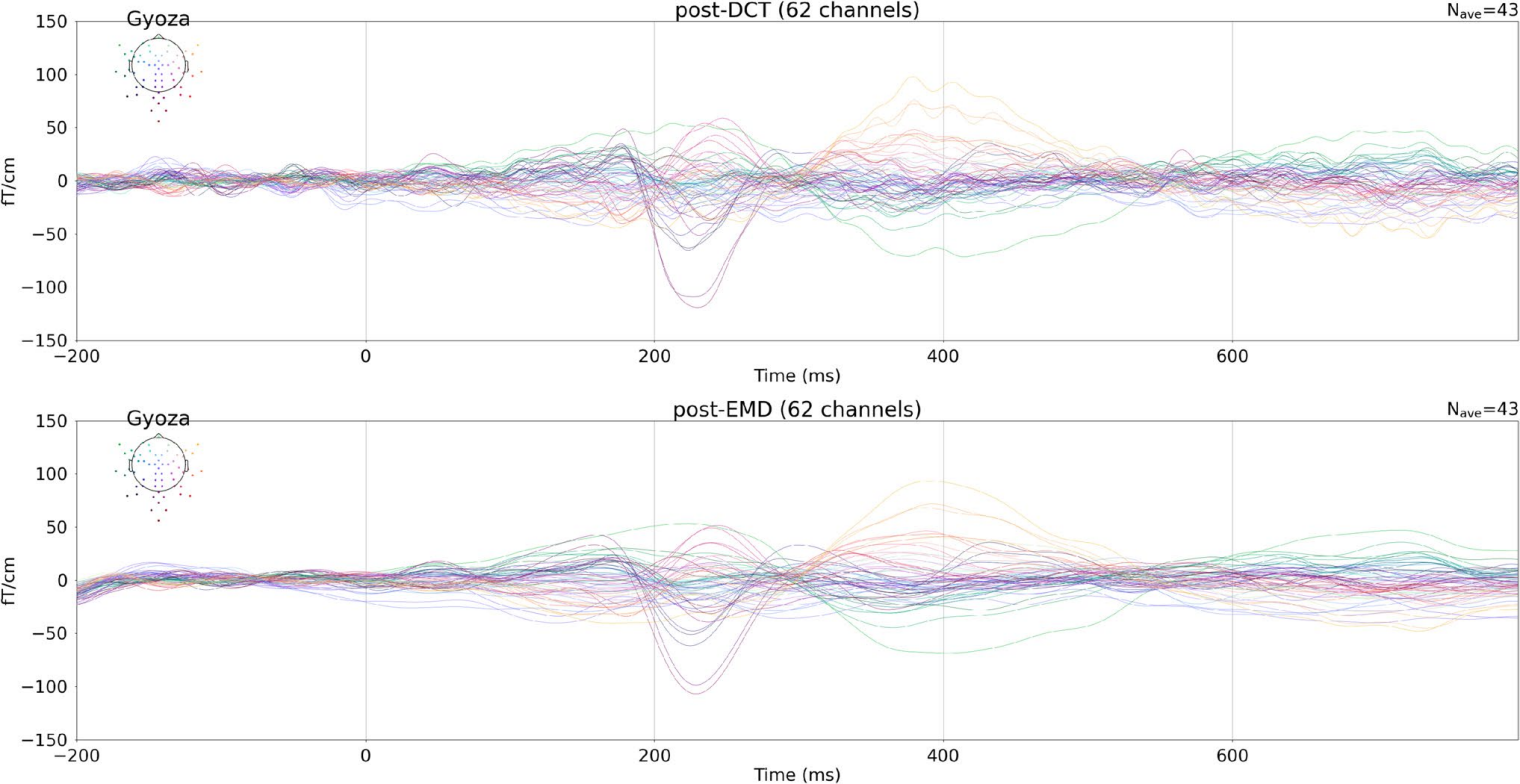
Example of the effects on the signals produced by the EMD filter. Upper panel shows grand average of a single type of event (Gyoza) using post-DCT data. Lower panel shows grand average of a single type of event using post-EMD data. The plots show 62 channels (all the good channels) color-coded to match the placement locations as shown in the upper left corner of each sub-plot. Gyoza refers to the image that was shown in this specific event. Upper right corner shows N_ave_ value, which refers to the number of averaged epochs in that sub-plot

As mentioned in section 0, classic BPF may produce strong distortions within the spectral bands close to the cutoff frequencies. To reduce distortion effects of the low-frequency noise filtration procedure, DCT-based filter was used. Whereas, for the high-frequency noise, EMD [32] based filter was used. The epochs were decomposed into their Intrinsic Mode Functions (IMFs). The IMF containing the highest frequency spectrum was removed from each epoch [Figure 4]. This procedure further reduced the Gamma frequency band along with the high-frequency distortions caused by the BPF.

#### Principal Component Analysis

The final step of the preprocessing procedure was Principal Component Analysis (PCA) filter. Through thorough investigation of our data, we realized that some of the main components contributing to the largest singular values, had no contribution in distinguishing between different event types. Therefore, they should be suppressed along with other undesired sources (e.g., noise). Due to this finding, we decided to use a Gaussian filter instead of rectangular when suppressing the singular values. PCA filtering was done on each epoch independently starting with normalization using z-score. Let us denote ${Ep}_{i}\in {\mathbb{R}}^{k\times n}$ as epoch number $i=\mathrm{1,2},\dots ,m$, where $k$ is the number of channels and $n$ is the number of samples.

1 $$\begin{array}{*{20}c} {\mu_{i} \; = \;\frac{1}{kn}\mathop \sum \limits_{c = 1}^{k} \mathop \sum \limits_{j = 1}^{n} Ep_{i} \left( {c,j} \right),} & {\sigma_{i} \; = \;\mathop {std}\limits_{c,j} \left( {Ep_{i} \left( {c,j} \right)} \right)} \\ \end{array}$$

where $c$ is the number of a channel and $j$ is the number of a sample. The epoch matrices are then normalized as follows:

2 $$\forall i,\;\ddot{E}p_{i} \; = \;\frac{{Ep_{i} - \mu_{i} }}{{\sigma_{i} }}$$

Next, a Singular Value Decomposition (SVD) is applied on the normalized epochs $\ddot{E}p_{i}$.

3 $$\ddot{E}p_{i} \; = \;U \cdot S \cdot V^{T}$$

Let us assume that the order of the singular values extracted from the diagonal of matrix $S$ is decreasing $\lambda_{1} \ge \lambda_{2} \ge \ldots \ge \lambda_{c}$. Denote $${\overline{\Lambda }} = \left[ {\begin{array}{*{20}c} {\lambda_{1} } & \cdots & {\lambda_{c} } \\ \end{array} } \right]^{T}$$. Gaussian filter function was defined as $\overline{G} = {\boldsymbol{N}}\left( {\frac{1}{4},\frac{1}{8}} \right)$, sampled at $\left[ {0,1} \right]$ (empirical values) with number of samples matching to the number of elements in ${\overline{\Lambda }}$. Meaning that $\overline{G}$ contains $c$ samples, the selection of the hyperparameters for $\overline{G}$ was based on the performance of the training session of our baseline machine learning model to which other models were later compared. The filtration is then applied by a simple Hadamard product marked as $\circ$. Resulting singular values were then used to create the filtered matrix $\tilde{S}$ of the same dimensions as matrix $S$. Note that singular values within $\tilde{S}$ are not necessarily in a decreasing order.

4 $$diag\left( {\tilde{S}} \right)\; = \;\overline{\Lambda } \circ \overline{G}$$

Here, ${\mathrm{diag}}\left( {\tilde{S}} \right)$ represents the diagonal of matrix $\tilde{S}$. All values off the diagonal are zeros. This procedure reduces the effects of large singular values and suppresses the effects of the small ones. Following, filtered epochs $\widetilde{Ep}_{i}$ are reconstructed using filtered singular values.

5 $$\begin{array}{*{20}c} {\widetilde{Ep}_{i} \; = \;U \cdot \tilde{S} \cdot V^{T} } \\ \end{array}$$

**Fig. 5:**
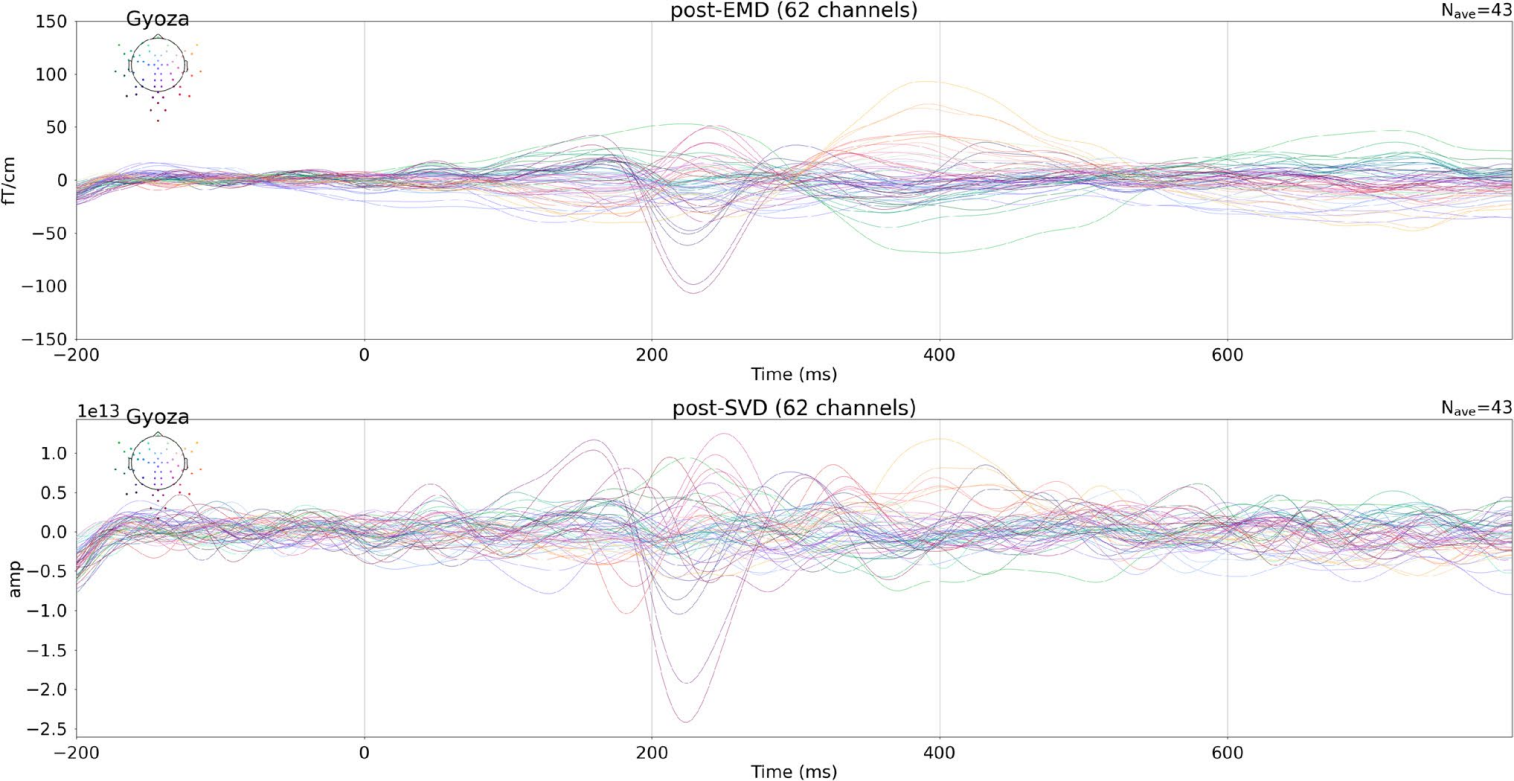
Example of the effects of PCA filter on the signals. Upper panel shows grand average of a single type of event using post-EMD data. Lower panel shows grand average of a single type of event using post-PCA data. The plots show 62 channels (all the good channels) color-coded to match the placement locations as shown in the upper left corner of each sub-plot. Gyoza refers to the image that was shown in this specific event. Upper right corner shows N_ave_ value, which refers to the number of averaged epochs in that sub-plot

The proposed filtration enhances informative patterns within the data, while suppressing the non-informative ones [Figure 5]. Even though when inspecting the resulted signals visually, post-EMD stage seems cleaner, classification accuracy of post-PCA is actually higher due to the enhancement of class-specific components.

### Combination’s Averaging

Combination’s Averaging (CA) is an approach developed to simulate averaging of sub-groups of epochs suitable for ML procedures. Our proposed method reduces the noise of the epochs while retaining more examples for ML models to use. Depending on the initial number of epochs, CA may even increase the size of the dataset. Thus, it is more suitable for smaller datasets than the sub-GA approach. The only difference between the classic sub-GA approach and the CA approach is how we select the sub-groups. Let us consider epochs $$\left[ {\begin{array}{*{20}c} {Ep_{1} } & \cdots & {Ep_{m} } \\ \end{array} } \right]$$, where $m$ is the number of epochs recorded for a certain event type. Let us denote $C$ as a set containing all of the sub-groups of epochs under the following constraints:

6 $$\begin{array}{*{20}c} {\left| {c_{i} \cap c_{j} } \right| \le b\; \Leftrightarrow \;|c_{j} |\; = \;|c_{j} |\; = \;a,\;\left| {c_{j} \cap c_{j} } \right| \le b} \\ \end{array}$$

where $\left| {c_{i} } \right|$ represents the number of epochs in sub-group “$i$”. Parameters $a \in { mathbb{N}}$ and $b \in { mathbb{N}}_{0}$ can be selected based on the constraints of the dataset. The idea of this method is to divide the epochs into sub-groups of $a$ epochs each, while the same epochs may also be used in other sub-groups. However, any two sub-groups may not have more than $b$ common epochs. This method can be seen as an extension of the classic sub-GA as they would be identical when $b = 0$. When creating the sub-groups $c_{i}$, it is recommended to select the parameters such that $\frac{b}{a} < \frac{1}{2}$, to reduce the potential of overfitting. The smaller that ratio is, the smaller the chances of any overfitting effects originating from the CA procedure. Reusing the same epochs for different sub-groups creates a certain level of statistical dependency between the resulting examples. This dependency level is directly affected by the ratio of $b$ to $a$. Such statistical dependencies may result in an overfitting of an ML model. However, as long as the mentioned ratio is low, it should not have any significant effects in terms of overfitting. Since the epochs are being reused in different sub-groups, it is mandatory to separate the “train” and the “test” datasets before the CA. The CA procedure should be performed on the “train” dataset separated from the “test” to avoid using any of the epochs from “test” in the combinations of the “train”. This is mandatory to avoid any test leaking. If an additional set, such as “validation” is required for the ML, it also should be separated before the CA procedure. The constraints for the set of sub-groups (6) allow for different possible sets that may vary in size. The calculation of the largest set can be quite difficult and may have an extreme computational load. Therefore, in our solution, we used a form of a greedy algorithm to locate some set of sub-groups (not necessarily the largest), that fits the constraints. The procedure was defined as follows:

1. Define $G$ as a set of all possible combinations of epochs in the dataset. Meaning that $$\left| G \right| = \left( {\begin{array}{*{20}c} m \\ a \\ \end{array} } \right)$$, where $m$ is the number of epochs in that dataset and $a$ is the number of epochs in each sub-group.
2. Select a random combination $C \leftarrow c_{i} \in G$.
3. Update group $$G \leftarrow G\backslash \left\{ {c_{j} \in G|c_{j} \cap c_{j} > b} \right\}$$.
4. Repeat steps (2) and (3) until $\left| G \right| = 0$.

This procedure allows for the creation of set $C$ that contains sub-groups of epochs under the constraints from (6) within reasonable computation time. To reduce the chances of selecting a small set of combinations, the procedure was performed ten times. The largest set of sub-groups was then selected from the resulting sets. Normally, this procedure should be done for each type of stimuli separately. It is recommended to prepare the selection scheme based on the number of different group sizes, rather than the number of different stimuli types. This is to reduce the number of different sized sets resulting from the procedure. If the initial number of epochs per event type is different (due to outlier removal, for example), then the resulting sets $C_{k}$ may also have different sizes. However, CA procedure allows for easy solution to assure that each set has the same number of examples whenever such constraint is important. We are already aware that the sets are most likely not of the largest possible size. Under these circumstances, having a few extra or a few less combinations makes little difference. Therefore, the solution becomes very simple. For each set, discard sub-groups with largest overlap size until it has the same size as the smallest set.

### Machine Learning

To classify different event types, four ML designs were used:

1. Classic Model (CM) – This is the classic approach of dividing the recorded epochs into “train” and “test” groups. Then, train and test the ML model using the sensor data from all the properly recorded channels. This is our baseline model for comparison.
2. Channel Selection Model (CSM) – This is the classic approach of dividing the recorded epochs into “train” and “test” groups. Apply ML procedure to conclude the highest contribution channels. Then, train and test the ML model using the sensor data only from channels with the highest classification contribution.
3. Combination’s Averaging Model (CAM) – This approach utilizes the CA procedure as explained in 0 to reduce the noise in the “train” dataset and increase its size. Then, train and test the ML model using the sensor data from all the properly recorded channels.
4. Channel Selection Combination’s Averaging Model (CSCAM) – This approach utilizes the CA procedure as explained in 0 to reduce the noise in the “train” dataset and increase its size. Apply ML procedure to conclude the highest contribution channels. Then, train and test the ML model using the sensor data only from channels with the highest classification contribution.

The models were used for in-subject classification. Meaning that training and testing were done for each subject independently. In all of the models, the data were reshaped from 3D tensors to matrices by concatenation of channels. Samples of the time segment 200ms–400ms (segment with highest variance) were used as ML features. Meaning that our feature vectors’ size was 100 (samples) multiplied by the number of used channels. The ML models tested as cores of our classification models were Support Vector Machine (SVM), Random Forest Classifier (RFC), and Hist Gradient Boosting Classifier (HGBC).

#### Classic Model

**Fig. 6:**
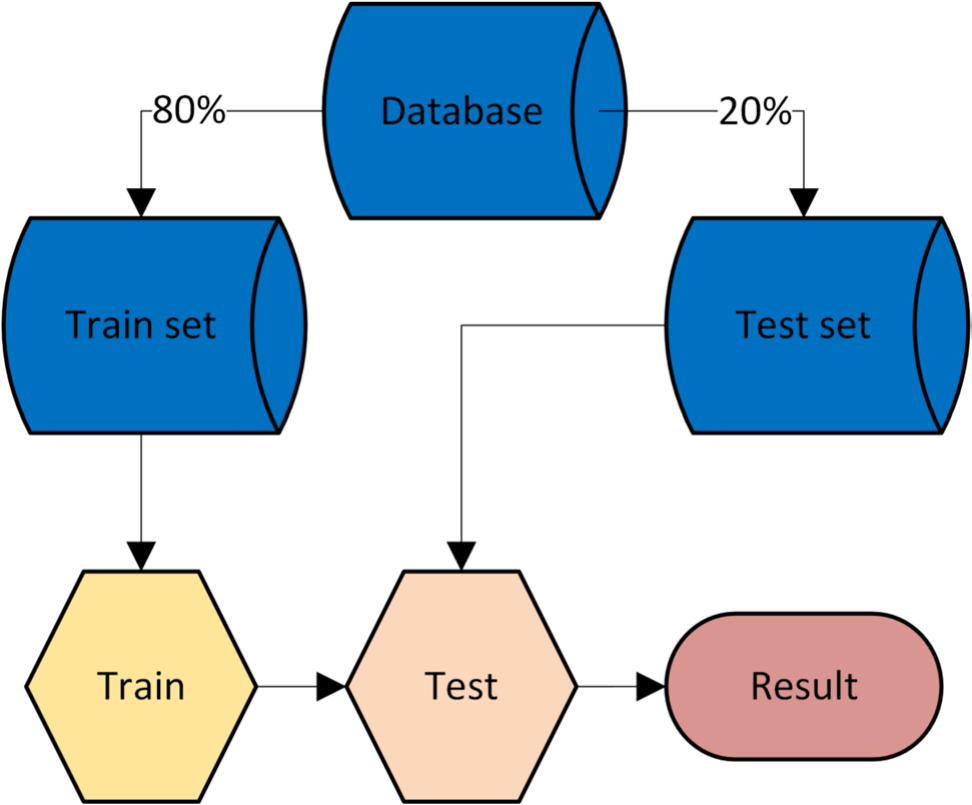
Flowchart of the Classic Model. Blue cylinders represent datasets, hexagonal blocks represent standard ML operations, and red oval is the result of the model

The CM, as the name suggests, is the classic approach of simply dividing the data into two sets, 80% for Train set and 20% for Test set. The selected model is trained on Train set and then tested on the Test set [Figure 6]. The main purpose of this model is to serve as a reference for the proposed algorithms.

#### Channel Selection Model

**Fig. 7:**
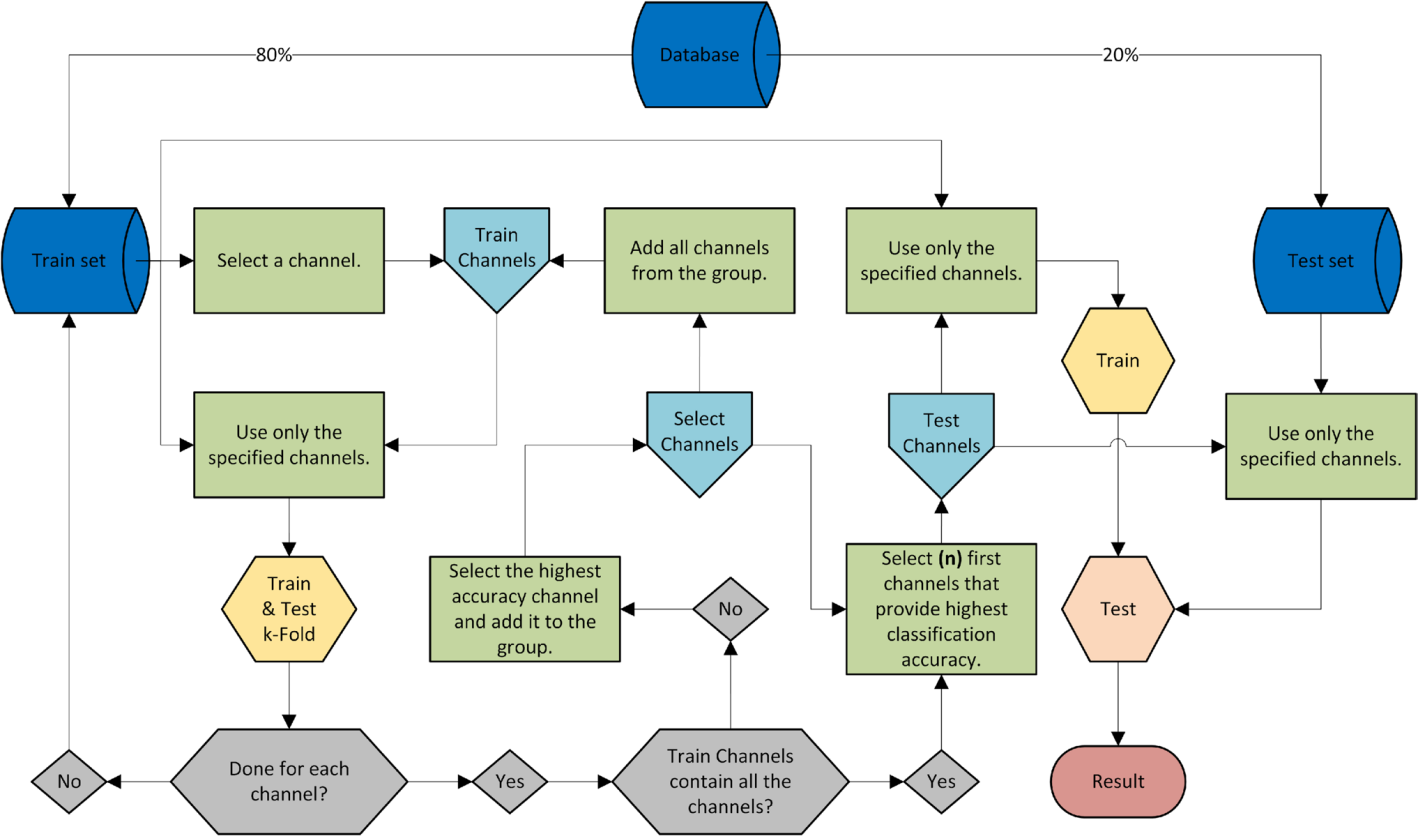
Flowchart of the cannel selection model. Blue cylinders represent datasets, hexagonal blocks represent standard ML operations, the trapezoid blocks represent some collection of items, the green rectangles represent a procedure to be performed, the gray elongated hexagon and the gray diamonds represent a condition check, and red oval is the result of the model

The CSM is similar to the CM in its concept, with an addition of channel selection procedure. This procedure attempts to learn the set of the most informative channels during the training session. The ML model is then trained using only the selected channels, while others are discarded [Figure 7].

The data were divided into Train and Test sets, 80 and 20%, respectively. Training procedure begins by selecting a single channel, then using a k-fold cross validation to calculate the average classification accuracy for that channel. In our case, based on the size of our dataset, “k” was selected to be 9. This procedure is done for each channel. Once complete, the channel providing the highest classification accuracy was selected. The procedure is then repeated for a pair of channels, using the one selected in the previous iteration together with each of the other channels. The channel that provided the highest accuracy when paired with the previous one was selected. This procedure was repeated in the same manner until all channels were used. Based on the received accuracies for each iteration, “n” channels were selected. Let us denote $c_{i}$ as the number of a channel selected at the iteration number $i$ and $m$ as the maximum classification accuracy index. The selected channel-set ${\Psi }$ is then defined as follows:

7 $$\begin{array}{*{20}c} {\Psi \; = \;\left[ {c_{0} , \ldots ,c_{m} } \right]} \\ \end{array}$$

Any channels not present in ${\Psi }$ were discarded from both Train and Test sets. The model is then trained and tested using the remaining channels. This procedure has two main purposes; First is to reduce the overfitting effects. Second is to reduce the size of the feature vectors.

#### Combination’s Averaging Model

**Fig. 8:**
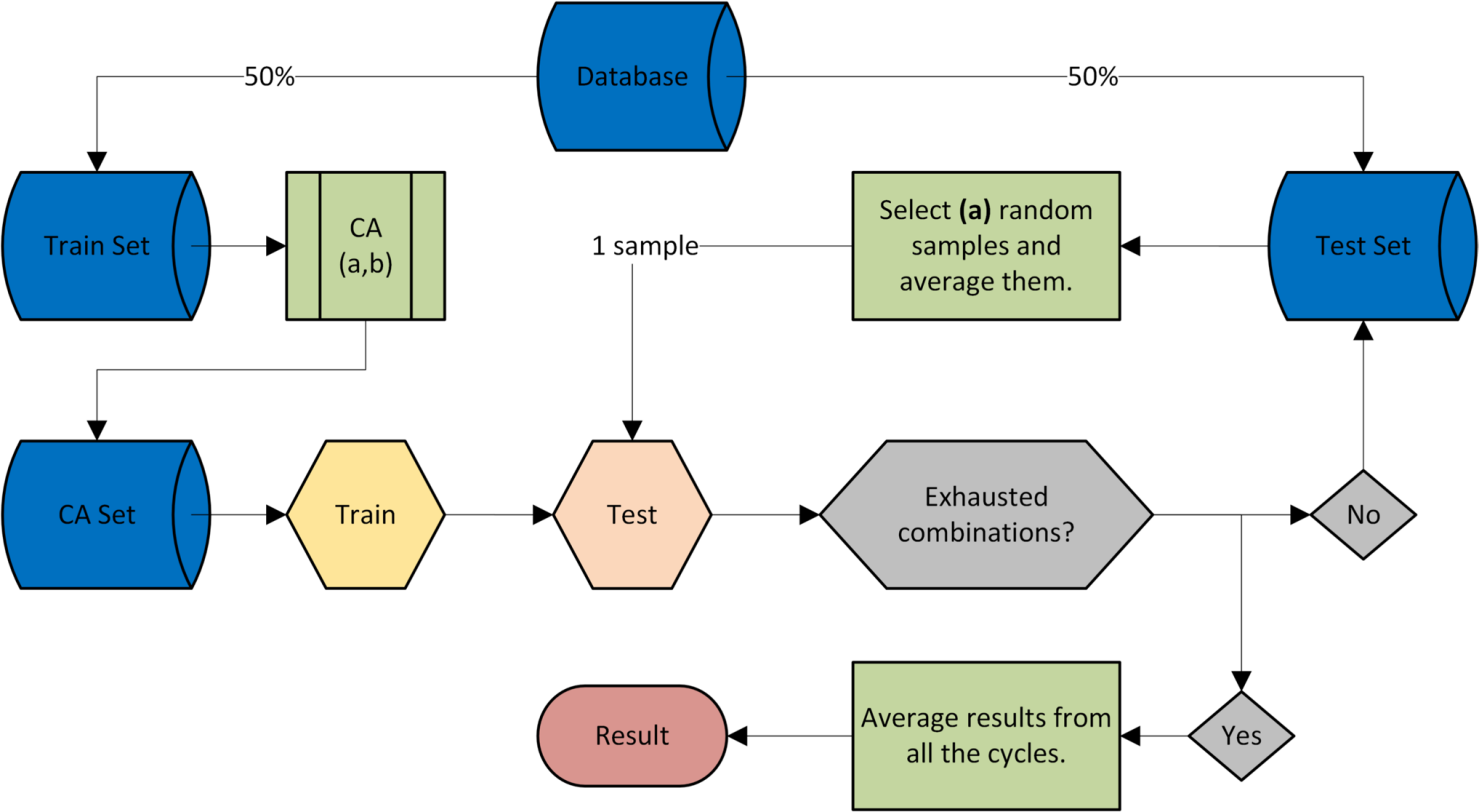
Flowchart of combination’s averaging model. Blue cylinders represent datasets, hexagonal blocks represent standard ML operations, the trapezoid blocks represent some collection of items, the green rectangles represent a procedure to be performed, the gray elongated hexagon and the gray diamonds represent a condition check, and red oval is the result of the model

The CAM utilizes the CA procedure as explained in 0. The dataset was divided into two groups, 50% for Train and 50% for Test. Train set was augmented using $\left( {a,b} \right) = \left( {5,2} \right)$ from (6). The model was then trained on the augmented CA set [Figure 8]. To preserve the relevance of the Test set, a “Leave k out” approach was used for testing. On each iteration, “$a$” random epochs were selected and averaged per stimuli, resulting in one averaged sample per stimuli type. The trained model was then tested on the selected example. The process was repeated until all combinations (with overlap less than “b”) were tested. The accuracy results were then averaged to calculate the overall classification accuracy.

#### Channel Selection Combination’s Averaging Model

**Fig. 9:**
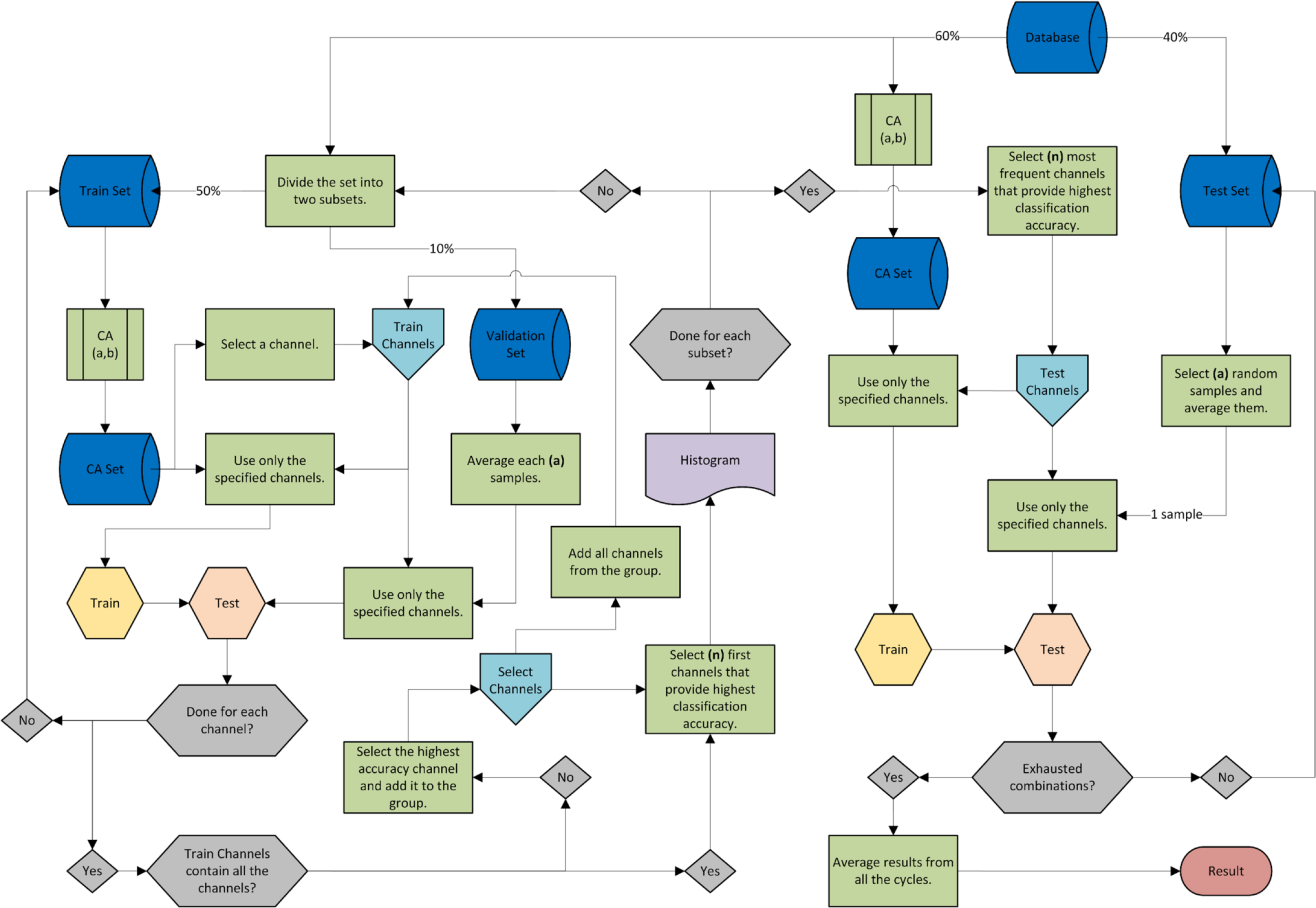
Flowchart of channel selection combination’s averaging model. Blue cylinders represent datasets, hexagonal blocks represent standard ML operations, the trapezoid blocks represent some collection of items, the green rectangles represent a procedure to be performed, the purple wavy rectangle represents statistics aggregation, the gray elongated hexagon and the gray diamonds represent a condition check, and red oval is the result of the model

The CSCAM is a combination of CAM and channel selection procedure. Due to the averaging step, channel selection is done in a “Leave k out” fashion. The dataset was divided into Train, Validation and Test sets, 50, 10, and 40%, respectively [Figure 9]. The ML model attempts to learn the most informative channels in the train session, similarly to the CSM.

Like in CAM, Train set was augmented using $\left( {a,b} \right) = \left( {5,2} \right)$ from (6). In the Validation set, each $a$ stimuli of the same type were averaged. Note that every set has the same number of stimuli for every type. In the same fashion as in CSM, a single channel was used in the first iteration. After each iteration, an additional channel was selected until all channels were used. Training was done on the Train set and testing on the Validation set. Next, following 7, most informative channels were selected and used to update the histogram. After which the sub-data (60%) were divided anew to Train and Validation sets and the whole process was repeated. This procedure continued until each of the epochs were used in the Validation set at least once. After each such iteration, the histogram of the most contributing channels was updated. Effectively creating a statistical map of channel contributions. The number of the selected channels was also preserved. Following, using the histogram, channels that contributed the most were selected. The number of selected channels from the histogram was the average of the selected channels used to update the histogram at each iteration. All other channels were discarded. Using only the remaining channels, the model was trained on the full Train set (60%) and then tested, on the so far untouched, Test set (40%). The testing procedure was identical to the one in CAM.

## Results

**Fig. 10:**
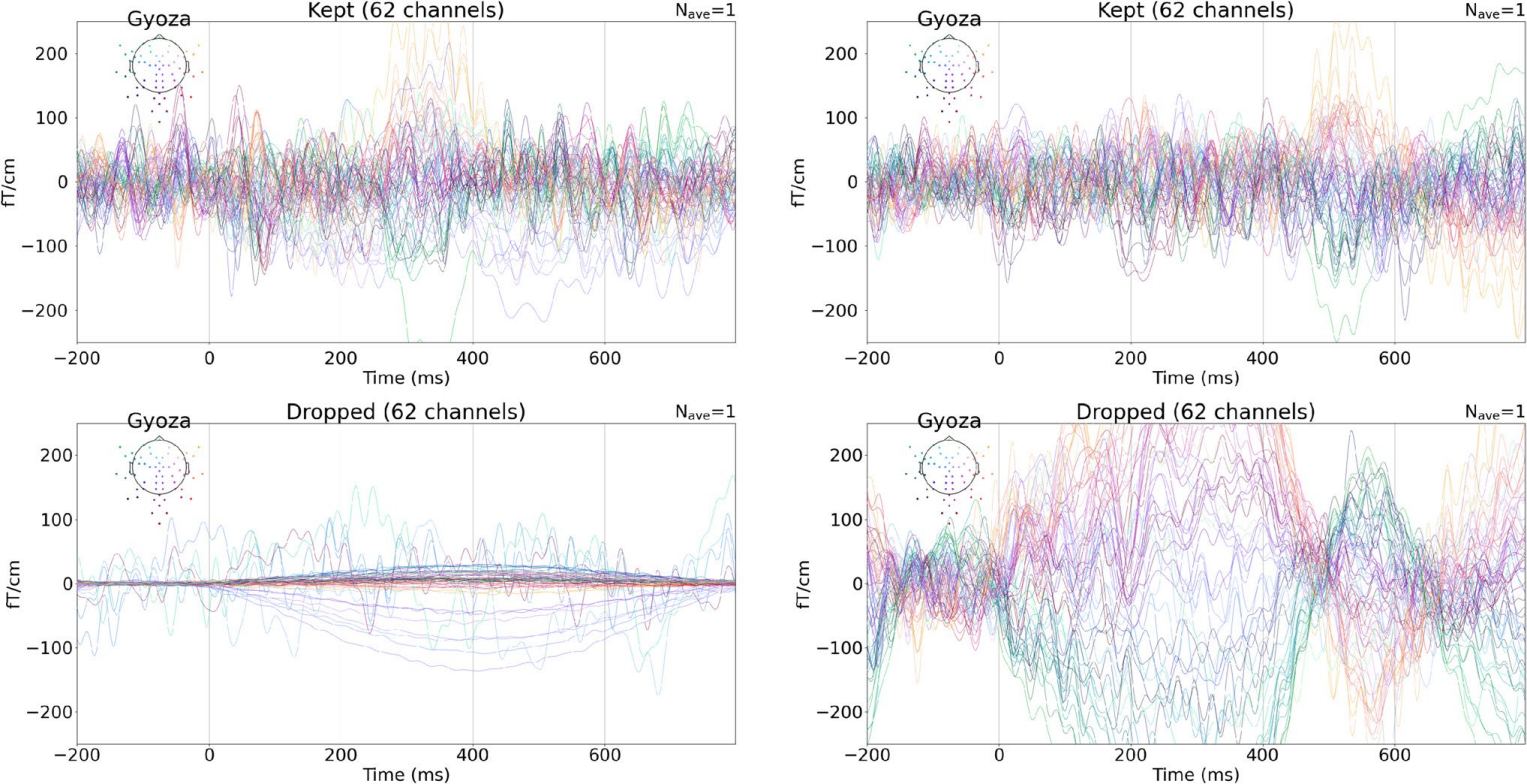
Example of the outlier epoch detection. Two sub-plots in the upper row show examples of the normal epochs from event type: Gyoza. Two sub-plots shown in the lower row, are examples of epochs detected as outliers (same event type: Gyoza). The plots show 62 channels (all the good channels) color-coded to match the placement locations as shown in the upper left corner of each sub-plot. Gyoza refers to the image that was shown in this specific event. Upper right corner shows N_ave_ value, which refers to the number of averaged epochs in that sub-plot

Using the proposed pipeline, the data were filtered, epoched, and cleared of outliers. Outlier detection results were visually inspected, which is one of the common ways to drop bad trials. The detection performed well, as can be seen in the example shown in Figure 10

Following the preprocessing steps, the data were used in four different ML designs as explained in 0 with CM serving as a baseline for comparison. Results of all subjects were aggregated to calculate the overall Receiver Operating Characteristic (ROC), Area Under the Curve (AUC) and confusion matrices for each model.

**Fig. 11:**
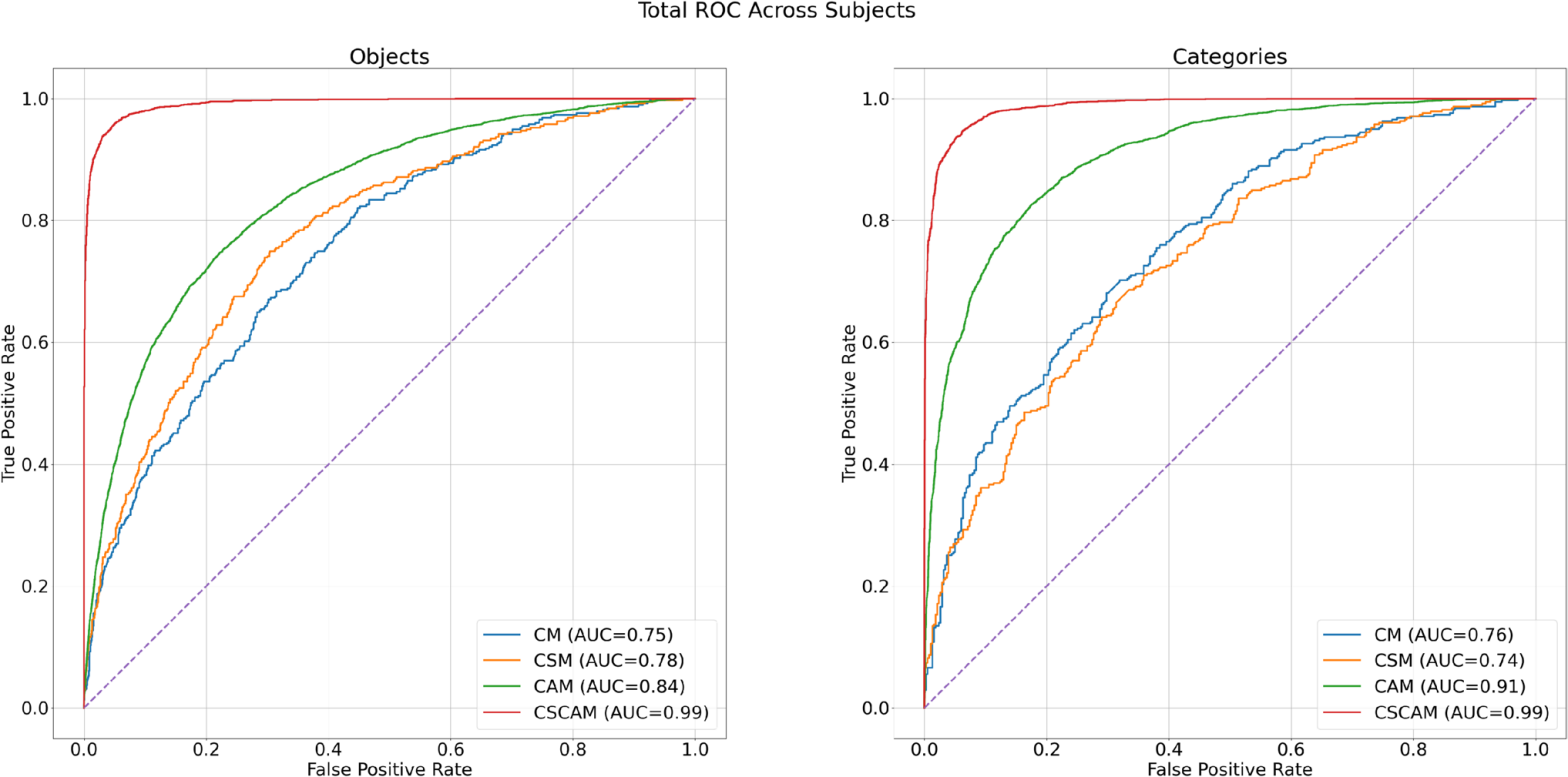
The figure shows ROC curves for each of the four models. Left sub-plot presents the multiclass case – Object classification. Right sub-plot shows the binary case – Category classification. AUC for each curve is shown in the bottom right corner of each sub-plot

**Fig. 12:**
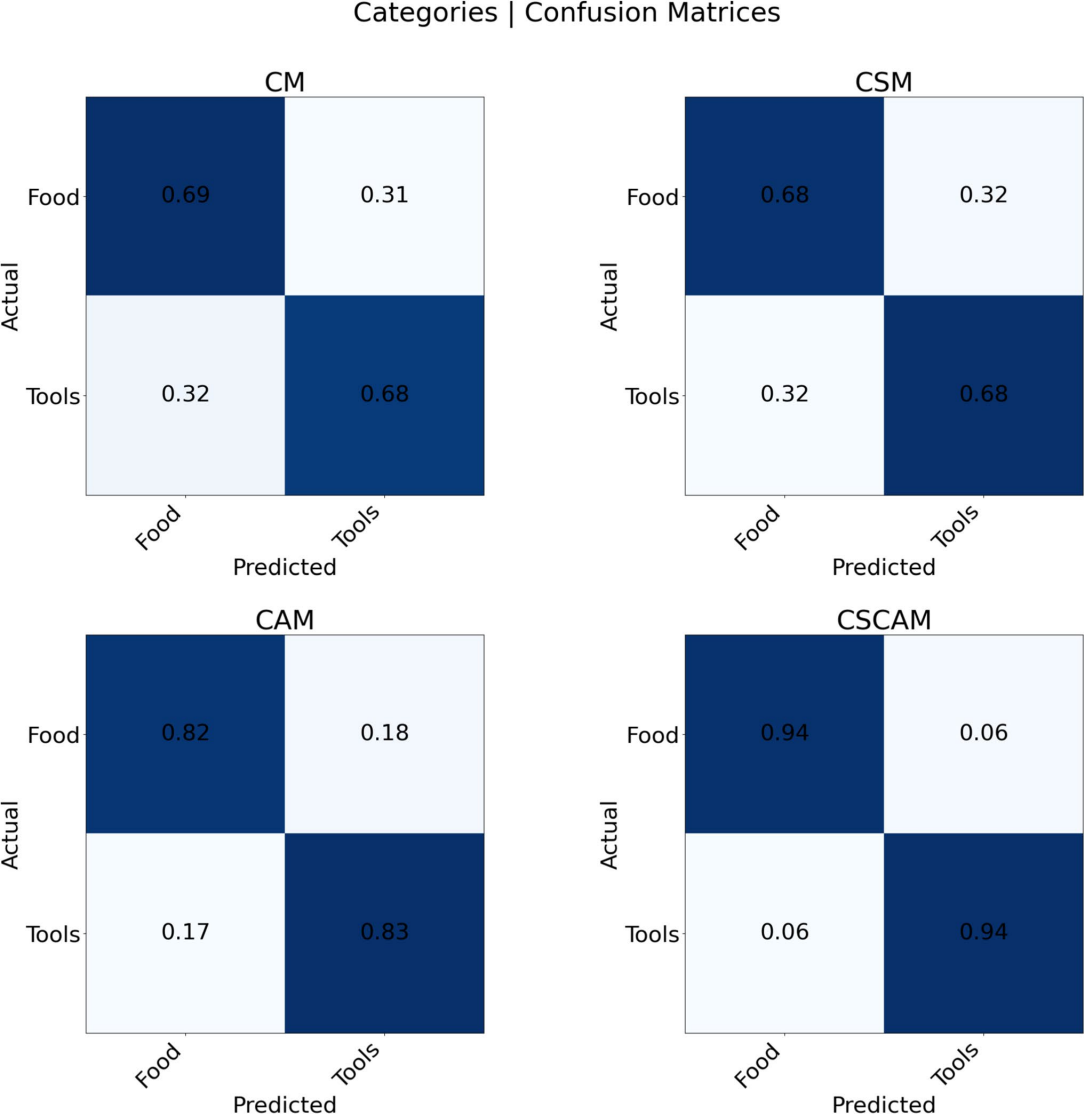
Confusion matrices of each model for binary case classification of categories

The baseline model—CM, achieved an AUC of about 0.75. Our CSM model has produced comparable results. Both models that used CA algorithm have performed better than the baseline. CAM has achieved an AUC of 0.84–0.91, while CSCAM has significantly outperformed all other models, reaching an AUC of 0.99 [Figure 11]. Category (binary) classification confusion matrices [Figure 12] show no clear affinity toward one class or another, in any of the models. About one-third of examples are mislabeled by CM and CSM. CAM mislabels less than 20% of examples, whereas CSCAM mislabels as little as 6% of epochs.

**Fig. 13:**
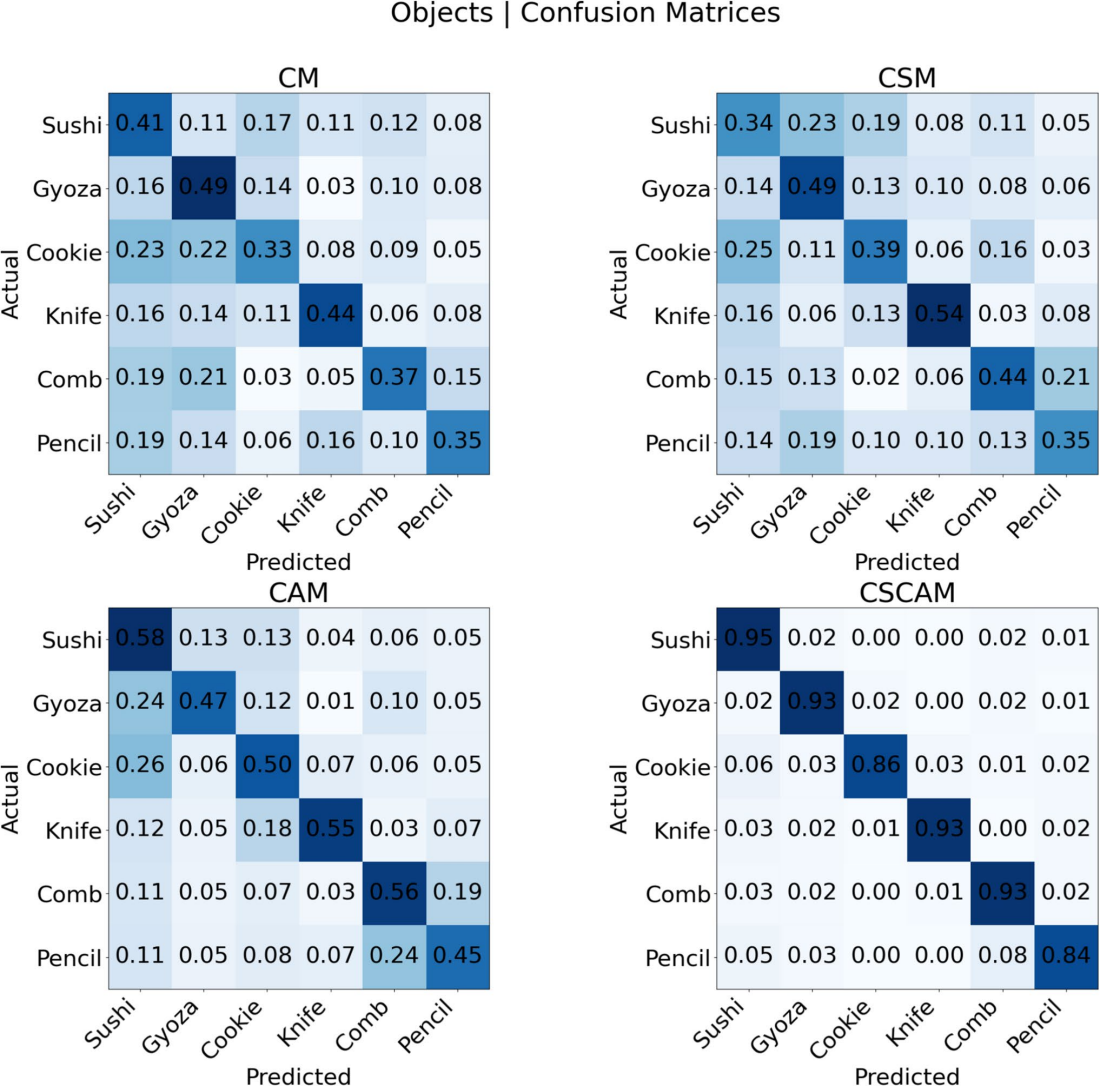
Confusion matrices of each model for multiclass case classification of objects

Object (multiclass) classification confusion matrices [Figure 13] are less balances than the binary case. CM shows objects being most frequently mislabeled as Sushi or Gyoza. CSM seems to reduce the tendency observed in CM, however, at the same time its mislabeling of Sushi is increased. CAM reduces the tendency toward Gyoza even more. While Sushi is still the most common mislabeling of objects, Gyoza is not much different from the other mislabeling cases. Additionally, bi-directional confusion is observed between Comb and Pencil. When examining CM and CSM carefully, similar confusion can be observed in these models as well, but may be a bit hard to notice do to relatively high mislabeling rate of other objects. CSCAM shows no affinity toward any of the classes when it comes to mislabeling, with only a minor mislabeling affinity of Pencil classified as Comb.

**Fig. 14:**
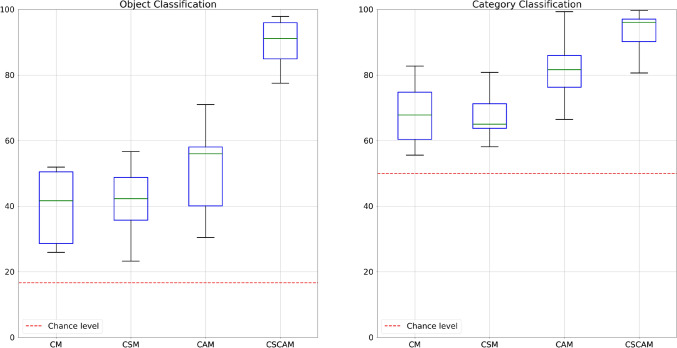
Left sub-plot shows object (multiclass) classification accuracy across subjects for each of the tested ML models. Right sub-plot shows category (binary) classification accuracy across subjects for each of the tested ML models. Red dashed line shows the chance level for each classification task

Figure 14 presents the boxplot of in-subject classification accuracies for object classification and for category classification by each ML design using SVM. Median accuracies for object classification after Interquartile Range (IQR) outlier removal were 41.67, 42.31, 55.94, and 91.10% for CM, CSM, CAM, and CSCAM, respectively. For category classification, they were 67.80, 65.00, 81.61, and 96.04% respectively [Figure 14]

The distribution of the classification accuracy was non-gaussian. Therefore, the statistical significance was tested using Wilcoxon Signed-Rank test, which is also considered to be a good choice for small datasets. In both Object and Category classifications presented in Figure 14, differences between CM and CSM were not significant, differences between CSM and CAM as well as differences between CAM and CSCAM were found significant (p < 0.05). There were no significant differences between SVM, RFC, and HGBC. However, the runtime of SVM was much shorter.

When inspecting the results on an individual level Table 1, it is evident that individual differences play a big role in the classification accuracy. Our results indicate that individual differences (Range) account for 20%–40% of differences in accuracy. The Root Mean Square Distances (RMSD) from median accuracy show the sensitivity of each model to the individual differences. For object classification, CM and CSM had comparable results, while CAM was more sensitive to individual differences. CSCAM was found to be the least sensitive to the individual differences out of the four models. For category classification, CM and CAM had comparable results, while CSM and CSCAM were found to be less sensitive, with CSCAM being the least sensitive once again Table 1

**Table 1:** Classification accuracy for each subject and each model

Object	Sub.2 (%)	Sub.3 (%)	Sub.4 (%)	Sub.5 (%)	Sub.6 (%)	Sub.8 (%)	Sub.10 (%)	Range	RMSD
CM	27.12	50.91	51.92	30.23	25.93	50.00	41.67	25.99	11.02
CSM	45.76	40.00	42.31	23.26	31.48	51.79	56.67	33.41	10.65
CAM	57.04	43.17	71.00	37.04	30.43	55.94	59.09	40.57	14.19
CSCAM	97.48	94.44	97.87	79.03	77.51	91.10	90.76	20.36	7.82

Category	Sub.2 (%)	Sub.3 (%)	Sub.4 (%)	Sub.5 (%)0	Sub.6 (%)	Sub.8 (%)	Sub.10 (%)	Range	RMSD
CM	67.80	74.55	82.69	55.81	55.56	75.00	65.00	27.13	9.42
CSM	62.71	74.55	80.77	58.14	64.81	67.86	65.00	22.63	7.56
CAM	81.61	80.67	99.32	71.85	66.47	85.80	86.11	32.85	9.83
CSCAM	96.32	92.86	99.65	80.65	87.57	97.72	96.04	19.00	6.91

Range column indicates the difference between the highest and lowest accuracies achieved by each model (i.e., individual differences). RMSD column indicates the root mean squares of distances from median accuracy (i.e., sensitivity to individual differences)

In terms of computational load, CM and CAM were comparable and had the best performance, with runtimes of less than 0.5m per subject. Note that CA’s combination selections were calculated in advance as they only require the total number of used epochs. Using this approach, CA procedure has little effect on the computational load of the model. CSM was significantly slower, with runtimes of up to 55m. Having the highest complexity, it is no surprise that CSCAM had the highest computational load, reaching runtimes of up to 22h per subject. Note that using the reported setup, all subjects were processed in parallel, using Python’s parallel processing tools. Therefore, the total runtime for the dataset (object or category classification) was the same as the total runtime for a single subject (23h).

## Discussion

In this study, we assess several different approaches to improve Neural Decoding accuracy of MEG data. As part of the pipeline, we have designed an automatic epoch-outlier detection algorithm that resolves the need for visual inspection of each and every epoch. One of the core algorithms presented in this work is the CA, data augmentation method, which was shown to be very effective, when used for ML purposes. The testing of multiple ML models has shown that averaging of epochs, together with careful channel selection may significantly improve the accuracy of Neural Decoding.

Our proposed preprocessing pipeline included several steps starting with downsampling of the signals to 500Hz. This step helps reduce the size of the feature vectors used in our approach. BPF is then applied to reduce the pass-band to 0.1Hz–40Hz as suggested in [31]. Due to the MEG device’s noise present below 1Hz, additional DCT-HPF was used with a cutoff frequency of 1Hz. Although DCT is also a part of Fourier methods which assume stationarity, it is well known that its performance on the non-stationary data is less destructive than most other similar methods when applied to low-frequency spectrum. It is a popular method for HPF applications in NS signals due to its relatively low distortions. It is often used to remove signals’ drift, which corresponds to the non-stationarity of the first order. It is also important to note that DCT’s performance creates relatively low distortions only in a low-frequency spectrum, when performing HPF. The distortions created in the high-frequency spectrum are usually comparable to other NS spectral techniques. Following this filtration procedure, the data were epoched into 1s segments, starting from 200ms before each stimuli onset. The proposed method of epoch-outlier detection was successful in detecting both high- and low-amplitude outliers [Figure 10]. Next, using EMD filter, an additional high-frequency component was removed. This helped to further reduce the noise and reduce the distortions from spectral filters within the data. A similar approach was suggested in [33]. The final step of the filtration process was PCA filter. There are multiple ways to apply PCA for filtering. Each one takes into account certain aspects of the data it is applied to. The most common application is the dimensionality reduction. Authors of [34] use PCA to improve their classification accuracy by retaining 99% of explained variance (i.e., rectangular window filter of singular values). Our PCA filter is the same type of filter as replacing small singular values with zeros. The only difference is in the filtering function. When zeroing the small singular values, it is the same as defining vector $\overline{{\boldsymbol{G}} }$ from (4) as a rectangular window. In our filter, instead of the rectangular window, a Gaussian window was used. This filter was used to suppress the non-informative components (small eigenvalues) and reduce the effects of the most dominant components (large eigenvalues) using a Gaussian window. Suppression of the small eigenvalues is a very common operation that was also utilized in [34]. It allows for the removal of signal components that hold no distinct information or have insufficient power for analyses. As for the large eigenvalues, it is not a very common practice to alter them. However, in some applications, the largest eigenvalues may correspond to some common phenomena that are of no interest for the target of the study. For example, in some of the neuroimaging techniques, largest eigenvalues may correspond to some systemic physiological factors other than brain activity. In such cases, reducing these components may improve the interpretability of the data, just as it did in ours. Note that our proposed PCA filter is just a minor modification of the commonly used approach. Each step of the preprocessing pipeline was assessed in terms of training classification performance of our baseline model, CM. Each preprocessing step has increased the classification accuracy of the training stage of CM. Effects of the preprocessing were not tested on our novel models (CSM, CSM, CSCSM). It would be interesting to test these steps on datasets from different MEG devices to investigate if any of the filtering contributions may be device specific. In general, there should be no issues applying this pipeline to any MEG dataset with some minor adjustments of the hyperparameters and frequency bands.

The four ML designs were tested with SVM, RFC, and HGBC. Although no significant differences were found between the results, the runtime of SVM was much shorter. Both CM and CSM provided accuracies of just above 40% for multiclass and around 65% for binary case classification, with no significant differences. However, depending on the application, one may be more suitable than the other. Training time of CM is much shorter than that of CSM. But at the same time, testing time of a single example is shorter for CSM due to significantly smaller feature vectors. In offline applications, where one would like to train and test the data only once, CM would suit better for its fast performance. In applications where training is done only once, but testing may be repeatedly required, CSM may be more suitable. One example of such case may be the real-time classification. The model can be trained beforehand, and then the classification is done in real time using smaller feature vectors, thus performing faster. The important information that can be provided by the CSM is the channels containing the neural information, which is important for decoding. This may allow to design devices with reduced number of channels that would perform just as well as the full headset. One interesting approach for the future study is to assess source localization accuracy when using only the channels which were selected by the model, after the removal of the channels which may have been used for noise reduction [35]. This may result in a higher accuracy of localization of areas responsible for objects or category recognition. Both CAM and CSCAM perform better in terms of classification accuracy but may not be applicable in real-time applications. This is mainly due to the requirement of averaging of multiple epochs. CAM produces higher accuracy results than CM and CSM and has a runtime comparable to that of CM if CA’s combinations were computed in advance. Since only the number of epochs is required to compute CA’s combinations, it is possible to do so beforehand and it was done so in this study. CAM’s improved accuracy can be explained by the reduction of noise when performing the averaging of multiple epochs. This was also shown in [20], where authors presented the classification accuracy based on the number of averaged epochs. Their results show a high improvement when averaging four to eight epochs. They have also shown that increasing the number of averaged epochs further does not produce much additional improvement. Thus, in our model, the number of averaged epochs was set to five with a maximal overlap of two. Although the averaging of multiple epochs is known to improve the interpretability of the neuroimaging data, the repeated usage of same epochs in different groups (the overlap) raises a concern of possible overfitting of the ML procedure. In our approach, to avoid any contamination or leakage of the test data, it was separated early on and was not used until the final testing stage. Under these conditions, any overfitting that may occur within the training stage cannot improve the results of the test stage. Therefore, any existing overfitting effects can only reduce the resulting accuracy of the proposed models. The individual differences affecting the classification accuracies similarly across models is also an indication that CAM and CSCAM are not affected by any significant overfitting effects that do not already exist within CM or CSM. Considering these factors, it is evident that the benefits of CSCAM procedure outweigh its limitations. This also shows that CA approach is successful in emulating the averaging of sub-groups of epochs and that the resulting augmented data are similar to an actual larger dataset that was reduced in size by the sub-GA procedure. CSCAM has a very long runtime when compared to the other three models, however, it is less sensitive to individual differences and its classification accuracy is the highest of the four models, reaching over 90%. We believe that there are several factors leading to these results. One is the reduction of noise within the epochs through averaging, just like in CAM. The second one is the selection of the most informative channels, thus reducing the irrelevant information within the feature vector, which in turn allows the ML model for a more accurate classification. The third reason is the size reduction of the feature vector, thus reducing the overfitting effects. The final reason is the increased dataset size allowing the model to learn from more examples. The remaining question would be: why don’t we see similar improvements when comparing CM and CSM? We believe that noise residing within the epochs are overwhelming ML models to the point where even the reduction of dimensionality and removal of irrelevant features are insufficient, especially considering the limited number of examples available for the model to learn from. Although our study mainly concentrated on an analysis of small datasets, most of the discussed techniques can be used in large datasets as well. It is important to note that when working with a large dataset, data augmentation may not be necessary, thus in CA, parameter $b$ may be set to 0. This will substantially reduce the computation cost, which is very high in the case of CSCAM. Next important question that is yet to be answered is which filtering techniques can be utilized to substantially reduce these in-epoch noise without the use of averaging. One of our future goals is to design such processing pipelines that would allow for high-accuracy classification of single epochs.

To conclude, in this work, the strengths and weaknesses of each filter were carefully considered. The possible distortions were taken into account, and the pipeline was designed with an intent to compensate for the distortions created by the NS procedures. This approach provides improved classification accuracy. In [33], the authors used similar filtration techniques and were able to perform a more accurate source localization, which further strengthens the validity of the proposed step. The CA method for real-data augmentation allowed for higher accuracy of classification. At the same time, the similarities between our findings and the ones in [20] show that this method does not create any unrealistic patterns within the data. Channel selection procedure can further improve the classification accuracy, but may not be very effective if the data exhibit strong internal noise. Further study should be done on the preprocessing techniques to find a substitute for epoch averaging. A performance comparison should be done using different datasets to assess any possible adjustments that may be beneficial for different MEG devices.
